# Synthesis and Catalytic Application of Silver Nanoparticles Supported on *Lactobacillus kefiri* S-Layer Proteins

**DOI:** 10.3390/nano10112322

**Published:** 2020-11-23

**Authors:** Patricia A. Bolla, Sofía Huggias, María A. Serradell, José F. Ruggera, Mónica L. Casella

**Affiliations:** 1 Centro de Investigación y Desarrollo en Ciencias Aplicadas “Dr. Jorge J. Ronco”—CINDECA (CONICET CCT-La Plata—UNLP—CIC), Calle 47 N° 257, B1900AJK La Plata, Argentina; pbolla@quimica.unlp.edu.ar (P.A.B.); sofi.huggias@hotmail.com (S.H.); jfruggera@quimica.unlp.edu.ar (J.F.R.); 2 Cátedra de Microbiología, Departamento de Ciencias Biológicas, Facultad de Ciencias Exactas, Universidad Nacional de La Plata (UNLP), 47 y 115 s/n, B1900AJK La Plata, Argentina; maserradell@hotmail.com

**Keywords:** silver nanoparticles, S-layer protein, DFT, catalytic activity

## Abstract

Research on nanoparticles obtained on biological supports is a topic of growing interest in nanoscience, especially regarding catalytic applications. Silver nanoparticles (AgNPs) have been studied due to their low toxicity, but they tend to aggregation, oxidation, and low stability. In this work, we synthesized and characterized AgNPs supported on S-layer proteins (SLPs) as bidimensional regularly arranged biotemplates. By different reduction strategies, six AgNPs of variable sizes were obtained on two different SLPs. Transmission electron microscopy (TEM) images showed that SLPs are mostly decorated by evenly distributed AgNPs; however, a drastic reduction by NaBH_4_ led to large AgNPs whereas a smooth reduction with H_2_ or H_2_/NaBH_4_ at low concentration leads to smaller AgNPs, regardless of the SLP used as support. All the nanosystems showed conversion values between 75–80% of *p*-nitrophenol to *p*-aminophenol, however, the increment in the AgNPs size led to a great decrease in *Kapp* showing the influence of reduction strategy in the performance of the catalysts. Density functional theory (DFT) calculations indicated that the adsorption of *p*-nitrophenolate species through the nitro group is the most favored mechanism, leading to *p*-aminophenol as the only feasible product of the reaction, which was corroborated experimentally.

## 1. Introduction

In recent years, nanomaterials have received great attention, due to their numerous applications in different areas of chemistry and biochemistry, and especially, for their interesting contributions in catalysis. Research on nanoparticles supported on biological supports is a topic of growing interest in nanoscience with catalytic applications, due to the great importance of these biological supports in the green biosynthesis of metallic nanoparticles. Catalysts based on noble metal nanoparticles (MNP) are highly attractive for their exceptional physicochemical properties compared to their bulk counterparts. The catalytic activity of metal nanoparticles at low temperatures is associated with their size and morphology [1,2]. Silver nanoparticles (AgNPs) have been particularly studied because they have lower cost and toxicity with respect to Pt, Au, Pd [3]. However, AgNps present several difficulties associated with their tendency to aggregation, oxidation, and low stability [4]. In addition, the catalytic properties of AgNPs are strongly influenced by their size, shape and nature and composition of the support [5]. In this sense, the support plays an important role in the stability of AgNPs, reducing their aggregation and improving their catalytic activity.

The synthesis of nanometric and subnanometric clusters emerges as an innovative tool for the development of new compounds, nanoelectronic components and catalysts. [6,7,8,9,10,11,12]. The quantization of the energy levels of a small metal cluster has characteristics like those of the isolated atom. This electronic behavior induces the appearance of unique catalytic properties. Recently, the use of biological or polymeric templates has made it possible to obtain metal clusters of controlled size [6,8]. Supramolecular interaction for the assembly of metal clusters is more efficient than synthetic protocols that require toxic and complex reagents. [6,13,14,15]. Noble metal nanoparticles have been successfully obtained on different supports (carbon nanotubes, surfactants, cyclodextrin, zeolites, MwCNT, etc.) [8,10,12,13,14,15,16,17,18,19,20,21,22]. The synthesis of nanomaterials with a controlled geometric structure is one of the challenges of chemistry and nanotechnology. Nature exhibits fascinating regular structures closely related to nanotechnology, allowing living organisms and their component structures to be candidates for use as a biotemplate. A biotemplate is a low-cost, easy-to-use and environmentally friendly alternative for the synthesis of nanostructures of a controlled size, because they have pores of regular and characteristics size. In addition, the biotemplate acts like a stabilizer of metal nanoparticles at room temperature. Biosystem machinery has higher precision compared to any artificial manufacturing technology. One of these biotemplates comes from S-layer protein (SLP), which is the outermost layer of some bacteria and archaea and is constituted by proteins or glycoproteins having a molecular weight of 40–200 KDa. This S-layer is made up of a set of individual proteins arranged in a plane, and one of its most extraordinary properties is its ability to assemble into a suspended or supported monomolecular crystal lattice. S-layer protein can be arranged with different lattice symmetries, oblique (p1, p2), square (p4) or hexagonal (p3, p6) [23]. The structure presents pores of identical symmetry and size, aligning the functional groups of the lattice in a defined way in order and orientation, which makes it a unique system for the assembly of the supramolecular structure. The monomers obtained by treatment with chaotropic agents can crystallize in a regular matrix that form plane sheets, open cylinders or spheres [24]. Mertig et al. were able to obtain highly ordered matrices of nanoparticles of metals of the Pt group onto an SLP of *Sporosarcine ureae* [25]. Also, SLP of *Bacillus sphaericus* were used to obtain a regular matrix with Pt and Pd nanoparticles located inside the pores of the matrix [26]. Our research group has previously obtained Pt and Ag nanoparticles supported on SLP of *Lactobacillus kefiri*, either isolated or forming an SLP/polymer complex [27,28]. Korkmaz et al. have succeeded in metallizing a recombinant SbsC S-layer protein with Pt to use it in nanobiotechnological applications [29].

Among the metallic nanoparticles synthesized using biological templates, those of silver nanoparticles (AgNPs) generate great scientific interest [30]. They are one of the most demanded nanoparticles due to their important uses in several areas, such medicine, pharmacology, biotechnology, engineering, energy and environmental applications [31]. In particular, AgNPs have been used in drug-delivery systems and diagnostic therapy, given their properties and low toxicity. By stabilizing AgNPs using proteins, it was possible to design systems to transport drugs into cells [30]. Nanoscale metallic silver is also of great interest in catalysis, due to its high performance in a variety of chemical reactions and its relatively low cost [32]. In the field of catalytic reductions, AgNP have been used in several hydrogenations, for instance, in the selective hydrogenation of dimethyl oxalate (DMO) to methyl glycolate. Dong et al. studied a catalyst based on AgNPs anchored inside the pores of amine-derivatized silica nanospheres, which was capable of hydrogenating DMO almost completely giving a selectivity to methyl glycolate of 97% [33]. Another important use of AgNPs as hydrogenation catalysts is the reduction of nitroarenes with NaBH_4_, a very important process both from the industrial and environmental point of view [34,35]. These and other investigations carried out using AgNPs have in common the fact that their catalytic activity depends on the size, shape and exposed facets of the nanoparticles. The design of AgNPs with characteristics that optimize their catalytic performance continues to be a great challenge. To help in this task, scientists have made use of DFT (density functional theory) calculations, through which the stability and architecture of the catalytic site generated by the nanoparticles can be evaluated, which in turn, added to the experimental knowledge of the system, will contribute to the “rational design of catalysts”.

A cluster having a finite number of atoms is ideal to study a catalytic process at an atomic level. The simplicity of the cluster allows a molecular-level knowledge of its performance to be obtained and it can be studied using computational methods to achieve a better understanding atom by atom. A cluster of loosely coordinated atoms is an especially active catalytic site, strongly affected by the support. The small size and the composition of the cluster is of central importance in catalytic activity. Clusters with only a handful of atoms have a high surface-to-volume ratio that offers exceptional chemical characteristics and high catalytic performance. Theoretical and computational methods play a central role in understanding and characterizing metallic clusters, being complementary to experimental methods [36]. Computational methods can explore intermediates and transition energy states that otherwise may be ignored in experimental studies. Theoretical simulation analysis of the different catalytic aspects (size, support, composition, intermediate reagents and products) in relation to the catalytic activity has been evaluated for different reactions involving AgNPs [36,37,38,39]. DFT is a widespread method, which can help us to understand the stability and electronic properties of nanoparticles, their adsorption modes on support surfaces, and to elucidate mechanisms and reaction rates for different reactions taking place on the catalyst surface, which together with experimental studies would allow the development of new catalysts, more efficient and in a more rational way [40].

In the present work, AgNPs supported on two S-layer proteins were synthesized and tested in the hydrogenation reaction of *p*-nitrophenol. Different reduction mechanisms of the nanocomposite were studied and their influence on the metallic nanoparticle size, as well as the influence of the different supports. Moreover, the adsorption of *p*-nitrophenol and *p*-nitrophenolate on Ag (111) and Ag (100) surfaces has been studied through DFT calculations, with the aim of understanding the interaction of the substrate with the catalyst surface.

## 2. Materials and Methods

### 2.1. S-Layer Protein Isolation

S-layer protein (SLP) was extracted from cultures of bacterial cells at stationary phase grown in Man–Rogosa–Sharpe (MRS) broth (Difco, Beauvais, France) at 32 °C for 48 h under aerobic conditions. Strains of *Lactobacillus kefiri* CIDCA 8348 and CIDCA 83,111 belonging to the collection of the Center for Research and Development in Food Cryotechnology (CIDCA, CONICET-CIC-UNLP) were used. The SLP isolation was performed using 5 M guanidine chloride (OmniPur®, Merck KGaA, Darmstadt, Germany) as previously described [28,41]. Briefly, bacterial cells were harvested by centrifugation at 16,000× *g* for 20 min at 4 °C and then washed three times with phosphate-buffered saline (PBS). The pellet was resuspended in a 10-fold volume of 5 M guanidinium hydrochloride in 0.05 M Tris-base (pH = 7.2) (J.T. Baker™, Avantor Inc., Radnor, PA, USA), stirred for 20 min at room temperature, and then centrifuged at 40,000× *g* for 30 min at 10 °C. This procedure was repeated twice, and the clarified supernatant was dialyzed against 10^−3^ M sodium ethylenediamine tetraacetate (EDTA) (Cicarelli, San Lorenzo, Santa Fé, Argentina) in 0.05 M Tris (pH = 7.8). The SLP’s solutions were adjusted to approximately 5 × 10^−6^ M and stored at 4 °C until use. Their quality was tested by sodium dodecyl sulfate-polyacrylamide gel electrophoresis (SDS-PAGE) (Bio-Rad Laboratories Inc., Richmond, CA, USA) and Coomassie blue staining (Pierce™, Thermo Scientific™, Waltham, MA, USA) [42].

### 2.2. Synthesis of S-Layer Protein (SLP)-Supported Ag Nanoparticles

Silver nanoparticles supported on two SLP extracted from *L. kefiri* CIDCA 83,111 (S_1_) and CIDCA 8348 (S_8_) were prepared. To do this, 47 µL of a 0.85 mg/mL AgNO_3_ solution was mixed with the corresponding SLP solution (1 mg/mL) for 24 h at 25 °C. Then, Ag reduction was carried out through three different methods. In the first method, AgNPs impregnated on each support were reduced under H_2_ flow at atmospheric pressure and 25 °C (hereinafter, referred to as Ag/S_1_G and Ag/S_8_G). In the second method, after a prereduction under the same conditions previously described, AgNPs deposited on both supports were treated with 0.02 M NaBH_4_ (PanReac AppliChem, ITW Reagents, Barcelona, Spain) (hereinafter referred to as Ag/S_1_R and Ag/S_8_R). Finally, a third pair of samples were reduced with NaBH_4_ (0.2 M) to obtain Ag/S_1_V and Ag/S_8_V systems. After each reduction method, the nanoparticles were centrifuged at 3500 rpm and the pellets were collected and repeatedly washed with 0.05 M Tris-base (pH = 7.2). Finally, the solids were resuspended in MilliQ water until use.

### 2.3. Characterization

The S-layer proteins (S_1_ and S_8_) were characterized by scanning electron microscopy using a TEM FEI Talos 200 microscope (Thermo Fisher Scientific, Waltham, MA, USA) operating at 80 kV, after staining the samples with 1% phosphotungstic acid for 40 sec and then air-drying them. The AgNPs were characterized using a Philips CM200-UT (LaB6) microscope (Amsterdam, The Netherlands) operated at 200 kV. This instrument also allowed performing energy dispersive X-ray (EDS) analysis. The samples were prepared by placing a drop of the AgNPs dispersion directly onto a carbon-coated cupper grid. The particle size distribution was determined counting more than 300 AgNPs using the ImageJ software (National Institute of Health, USA). Particles were considered spherical and the number (*d_n_*), surface (*d_s_*) and volume (*d_v_*) mean diameters were calculated according to the following formula:

(1) $$d_{p-q}=\frac{\sum n_{i}d_{i}{}^{p}}{\sum n_{i}d_{i}{}^{q}}$$

where *n* is the number of particles with di diameter, and *p-q* were 1–0, 3–2 and 4–3 for *dn*, *ds* and *dv*, respectively. Metal dispersion (*D*) was calculated with the following equation:

(2) $$D=\frac{6M_{Ag}}{\sigma \rho_{Ag}}\left(\frac{\sum n_{i}d_{i}{}^{2}}{\sum n_{i}d_{i}{}^{3}}\right)$$

where *M_Ag_* is the molar mass and *ρ_Ag_* is the density of silver, and *σ* is the area occupied by 1 mol of Ag (5.2675 × 10^4^ m^2^/mol).

### 2.4. Catalytic Test: p-Nitrophenol Reduction

The performance of Ag/S_1_G, Ag/S_8_G, Ag/S_1_R, Ag/S_8_R, Ag/S_1_V and Ag/S_8_V catalysts was evaluated using the reduction of *p*-nitrophenol (*p*-NP) with NaBH_4_ as a model reaction, following the procedure previously reported by our research group [41]. Briefly, *p*-NP presents an absorption maximum at 317 nm that shifts to 400 nm due to the predominance of the *p*-nitrophenolate ions because of the increased alkalinity after addition of NaBH_4_. The progress of the *p*-nitrophenolate reduction into *p*-aminophenolate is followed by the decrease in the absorbance of this band. The reactions were carried out in situ using a quartz spectrophotometer cell (1 cm) as a reactor, mixing 0.3 mL of the catalyst with 0.5 mL of 5 × 10^−4^ M *p*-NP, 0.02 M NaBH_4_, and 1.5 mL MilliQ water. The reduction was performed at atmospheric pressure and room temperature in a Cintra 20 Spectrophotometer (GBC Scientific Equipment, Braeside, Victoria, Australia). Absorption spectra in a range of 200 to 600 nm were registered periodically for approximately 60 min. Conversion of *p*-NP (*X*%) was calculated as follows,

(3) $$X\%=\frac{\left(A_{o}-A_{t}\right)100}{A_{o}}$$

where *A_0_* and *A_t_* are the absorbances at zero and t time (min), respectively.

The studied reaction has been kinetically modeled by several authors, concluding that it presents a Langmuir–Hinshelwood type kinetics [2,43]. Since NaBH_4_ was placed in excess respect to *p*-NP, the reaction kinetics were treated as pseudo-first-order regarding *p*-NP concentration. Thus, the apparent rate constant (*K_app_*) was obtained using Equation (4), where *C_t_* and *C_0_* are the concentrations at time *t* and time 0, respectively.

(4) $$K_{app}t=-ln\left(\frac{C_{t}}{C_{0}}\right)=-ln\left(\frac{A_{t}}{A_{0}}\right)$$

Turnover frequency (TOF) was calculated using Equation (5):

(5) $$TOF=\;\frac{{\mathrm{mol}}_{p-\mathrm{NP}}\;K_{app}M_{Ag}}{m_{Ag}\;}$$

where mol_p-NP_ are the mol of *p*-NP, m_Ag_ is the mass of Ag in the catalyst, *M_Ag_* is the atomic mass of silver and *K_app_* is the apparent rate constant.

### 2.5. Theoretical Calculations

DFT calculations were done using the Quantum Espresso package (GNU General Public License, Quantum EXPRESSO Foundation) [44,45]. Ultra-Soft pseudopotentials with scalar relativistic correction generated by the Rappe–Rabe–Kaxiras–Joannopoulos method (RRKJUS) and a PBE density functional were used [46]. The cutoff energy chosen was 250 Ry. The threshold for self-consistency was 1x 10^−6^ eV. Brillouin zone integration was approximated using the Monkhorst–Pack scheme [47] with 1 × 1 × 1 *k*-point in all calculations. The vacuum between the slabs was set at 25 A. A *p*(4 × 4) supercell with three metal layers was used. The first two layers were fixed at the bulk position and the last one was allowed relaxing when the substrate is adsorbed. The geometry relaxation was undertaken using the BFGS quasi-Newton algorithm until the forces on each atom were less than 10^−5^ eV/A and the energy difference of consecutive steps was less than 10^−5^ eV.

Adsorption energies $E_{ads}$, were calculated from total energies according to:

(6) $$E_{ads}=E_{syst}-\left(E_{Ag}+E_{\;}{}_{subst}\right)\;$$

were *E_syst_* is the energy of substrate adsorbed on the metallic surfaces (both (111) and (100) faces), *E_Ag_* is the energy of the clean surface and *E_subst_* is the energy of each free substrate in the vacuum. With the definition in Equation (6), a negative $E_{ads}$ means a stable adsorption of the substrate on the surface.

## 3. Results and Discussion

### 3.1. Isolation and Characterization of the S-Layer Protein Supports

**Figure 1:**
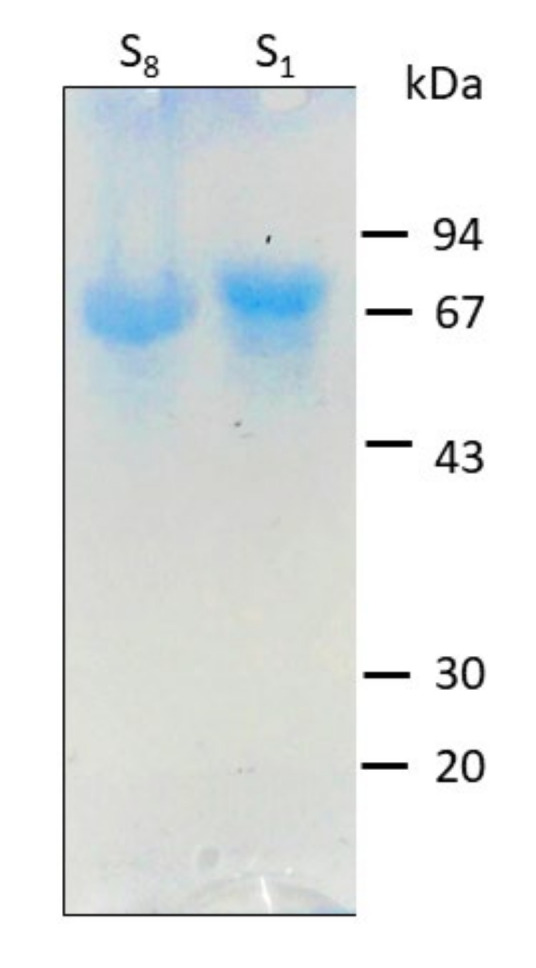
Sodium dodecyl sulphate-polyacrylamide gel electrophoresis (SDS-PAGE) of *L. kefiri* CIDCA 83,111 (S_1_) and CIDCA 8348 (S_8_). Molecular mass markers are shown on the right (kDa).

It has been previously reported that the SLP from different strains of *L. kefiri* showed an apparent molecular weight ranging from 67 to 71 kDa and a calculated pI of 9.6 [48,49]. The S_1_ and S_8_ SLPs are glycosylated, coming from strains exhibiting distinct aggregating capacity, and present differences both at the level of their amino acid sequence and composition [49,50,51]. The presence of the glycosidic chains in these proteins may help to understand their behavior towards the obtention of well-stabilized AgNPs. SDS-PAGE analysis of S_1_ and S_8_ SLPs showed a unique band corresponding to a molecular weight between 67–69 kDa (Figure 1).

**Figure 2:**
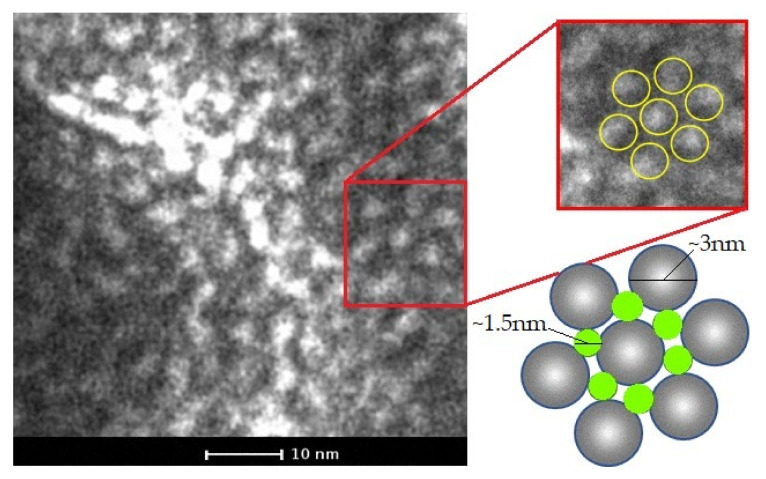
Transmission electron microscopy (TEM) micrography of S-layer protein (SLP). Zoom and representation of *p6* arrangement.

SLP subunits are generally arranged in the form of symmetrical networks, with oblique (*p2*), square (*p4*) or hexagonal (*p6*) cavities, generating a characteristic two-dimensional arrangement. This property has made it difficult to obtain SLP crystals. Indeed, we have tried to crystallize SLPs in order to carry out XRD studies, but we have not yet succeeded. Figure 2 shows this characteristic two-dimensional regular arrangement for the case of the S_8_ protein, obtained by TEM. The size of the subunits arranged in a hexagonal arrangement (*p6*) is around 3 nm with pores of around 1.5 nm. The bidimensional arrangement of the SLPs could direct the synthesis and disposition of Ag nanoparticles making it an excellent support.

### 3.2. Synthesis and Characterization of Silver Nanoparticles (AgNPs)

Obtaining metallic nanoparticles with interesting catalytic characteristics requires careful control of their growth during the reduction process. Thus, for example, Piñero et al. [52] have studied the relationship between size and general behavior in terms of intensive physicochemical properties of the particles, according to the length scale of the system. These authors define particles according to their size as: atoms (less than 0.3 nm), small clusters (from 0.3 to 1 or 2 nm), large clusters (from 1 or 2 to 4 nm), NPs (from 4 to 100 nm) and bulk when the size is greater than 100 nm.

**Figure 3:**
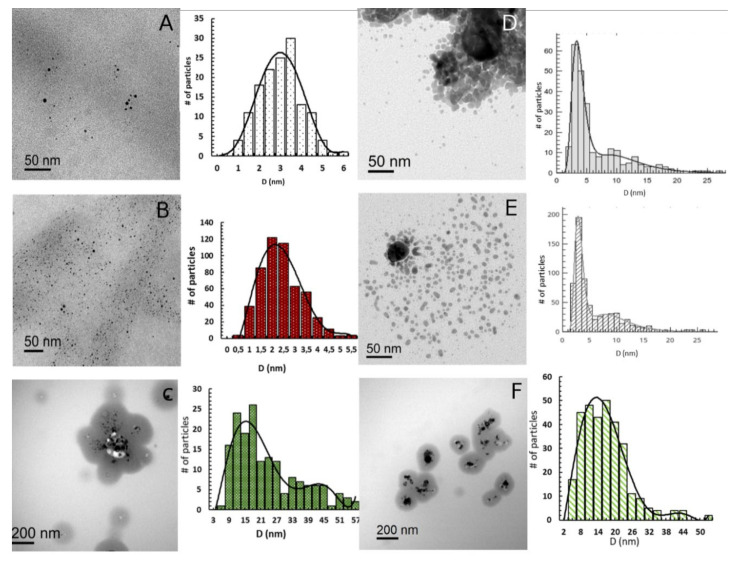
TEM bright field images of Ag/S_1_ and Ag/S_8_ samples, with their respective size distribution histogram. The histogram was obtained counting 300 NPs on average. (**A**) Ag/S_8_G, (**B**) Ag/S_8_R, (**C**) Ag/S_8_V, (**D**) Ag/S_1_G, (**E**) Ag/S_1_R and (**F**) Ag/S_1_V.

The systems presented in the present work can be considered formed by either clusters or NPs, according to the classification mentioned in the previous paragraph. TEM images showed that both S-layer proteins employed as templates, S_1_ and S_8_, are decorated by Ag nanoparticles with most of them evenly distributed, although some small aggregates can also be observed (Figure 3). Both Ag/S_1_G and Ag/S_1_R exhibited a bimodal AgNPs size distribution. The particle size distribution observed for the Ag/S_1_G system showed a first mean value at *d_n_* = 3.3 nm (*d_s_* = 4.6 nm; *d_v_/d_n_* = 1.51) and the second one at *d_n_* = 10.2 nm (*d_s_* = 12.5 nm, *d_v_/d_n_* = 1.43) (Figure 3D). On the other hand, the Ag/S_1_R system showed an ensemble of round particles with *d_n_* = 3.20 nm (*d_s_* = 6.6 nm; *d_v_/d_n_* = 2.43; Figure 3E), with a broader size distribution. The second peak represents particles having a mean size of *d_n_* = 9.1 nm (*d_s_* = 11.2 nm; *d_v_/d_n_* = 1.43). When considering the AgNPs obtained by reducing the silver promoter with NaBH_4_ (Ag/S_1_V) a monomodal distribution with *d_n_* = 15.81 nm (*d_s_* = 25.24 nm; *d_v_/d_n_* = 1.94) was observed (Figure 3F).

S8-supported silver nanoparticles obtained using either hydrogen (Ag/S_8_G) or hydrogen plus small amounts of NaBH_4_ (Ag/S_8_R) as reducing agents showed smaller particle sizes than their S1-supported counterparts. The mean particle size of Ag/S_8_G and Ag/S_8_R systems were *d*_n_ = 2.77 nm (d_s_ = 3.65 nm; *d_v_/d_n_* = 1.4) and *d_n_* = 1.9 nm (*d_s_* = 2.65 nm; *d_v_/d_n_* = 1.6), respectively (Figure 3A,B). Regarding Ag/S_8_V, a particle size of *d_n_* = 21.01 nm (*d_s_* = 36.38 nm; *d_v_/d_n_* = 2.03) was observed, being the biggest particles of all the studied ones (Figure 3C).

Summarizing, silver nano particles of different sizes could be prepared changing the reducing agent. While the drastic reduction by NaBH_4_ led to large AgNPs, a smoother reduction with H_2_ or H_2_/NaBH_4_ in low concentration led to smaller particles, regardless of S1 or S8 being used as template. AgNPs supported on S1 protein and then reduced in mild conditions show a bimodal distribution with large clusters and NPs while AgNPs supported on S8 showed small and large clusters. The samples Ag/S_1_V and Ag/S_8_V exhibit only NPs according to this definition.

**Figure 4:**
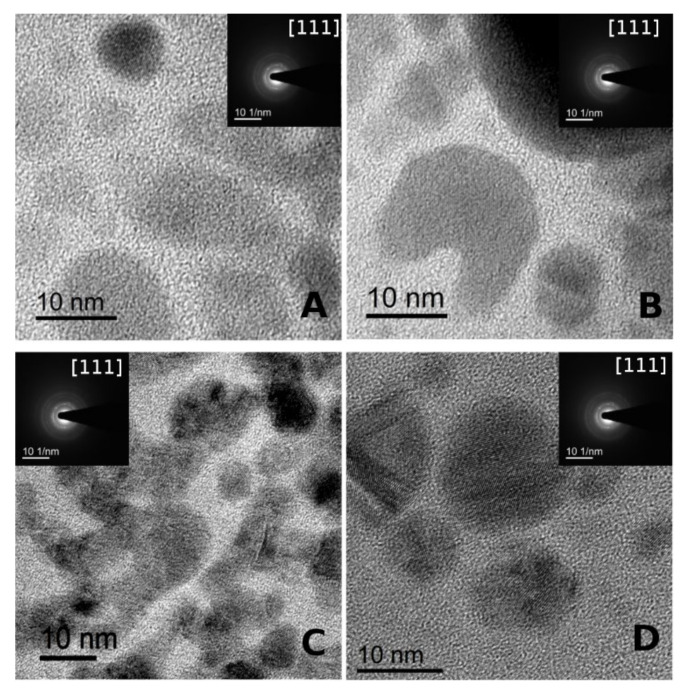
High-resolution transmission electron microscopy (HR-TEM) image of silver nanoparticles (AgNPs) and their fast Fourier transform (FFT). (**A**): Ag/S_1_G (**B**): Ag/S_1_R (**C**): Ag/S_8_G. (**D**): Ag/S_8_R.

High-resolution transmission electron microscopy (HR-TEM), fast Fourier transform (FFT), and selected area electron diffraction (SAED) of the AgNPs are shown in Figure 4. The presence of AgNPs on S-layer protein supports was confirmed by EDS (not shown). Moreover, the AgNPs present crystalline structure evidenced from crystalline planes observed in each diffraction pattern (Figure 4A–D).

### 3.3. Catalytic Test: p-Nitrophenol Reduction

Nitroaromatic compounds are widely used in the manufacture of pharmaceuticals, pigments, dyes, plastics, pesticides and fungicidal agents, explosives, and industrial solvents [53]. Among them, *p*-nitrophenol (*p*-NP) has been classified as a priority pollutant by the United States Environmental Protection Agency (EPA) [54] because it is stable in the environment and resists biodegradation. Therefore, it is highly desirable to develop an ecologically clean technology to treat such compound in an aqueous medium. As a consequence, the reduction of *p*-NP with sodium borohydride to produce *p*-aminophenol (*p*-AP) is of industrial and environmental importance besides being the typical reaction used to investigate the catalytic properties of metallic nanoparticles. Various metallic nanoparticles such as Ag, Au, Cu, Pt and Pd supported on different substrates such as dendrimers, polyelectrolytes, biological cells, etc., have been used as catalysts for similar reactions [55]. However, the addition of NaBH_4_ has been reported to destroy colloidal stability, causing catastrophic aggregation and drop of the catalytic activity [56].

The hydrogenation of *p*-NP to *p*-AP reaction can be considered a pseudo-first-order reaction. Thermodynamical calculation showed that the hydrogenation of *p*-nitrophenol is a feasible process. However, it is kinetically restricted and does not occur even after 24 h without a catalyst [2]. Catalytic activities of Ag/S_1_GAg/S_1_R,Ag/S_1_V,Ag/S_8_G,Ag/S_8_,R and Ag/S_8_V were investigated using the reduction reaction of *p*-PN to *p*-AP at room of temperature.

The disappearance of the yellow color corresponding to the decrease of the absorbance band at 400 nm was measured by ultraviolet–visible (UV−Vis) spectroscopy.

**Figure 5:**
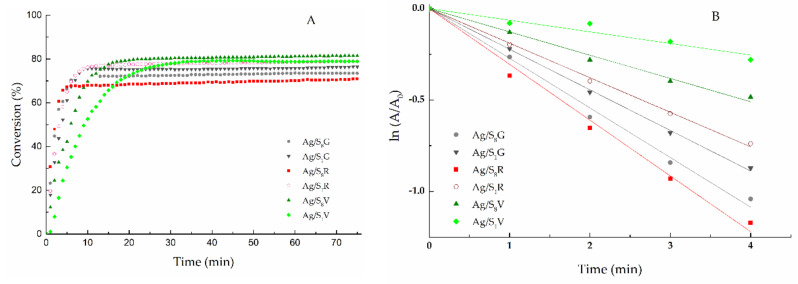
Catalytic activity of the different bionanocatalysts. (**A**) Conversion vs. time. (**B**) ln (*A_t_*/*A_0_*) vs. *t*. The *K_app_* values were obtained using Equation (4).

When Ag nanoparticles are added to the reaction cuvette, the peak at 400 nm disappears in a few minutes. The *p*-AP is the only reaction product, as is demonstrated by the presence of an isosbestic point, as has been previously reported for similar systems [41]. Figure 5 shows the time-dependent conversion curves for all the studied systems. Conversion values between 75 and 80% of *p*-NP were observed for all the Ag NPs tested, regardless of the average particle size of the NPs. Unlike these results, Fenger et al. [57] found that a set of medium-sized Au nanoparticles (10–13nm) were the most efficient, exhibing a higher catalytic activity than smaller and larger particles. In this paper, non-significant differences in conversion values were observed, regarding the size of silver nanoparticles. Probably, this is due to a higher accessibility of the reagent at the active site, given the structural order provided by the S-layer protein. Nevertheless, the values of the apparent rate constants seem to be influenced by the size of the nanoparticles, as illustrated in Figure 5B.

The apparent rate constant (*K_app_*) was higher for Ag/S_8_R (18.5 h^−1^) and Ag/S_8_G (17.2 h^−1^) than for Ag/S_1_G (16.8 h^−1^) and Ag/S_1_R (13.7 h^−1^) catalysts. This result can be explained by the smaller size of Ag/S_8_G (*d_s_* = 3.5 nm) and Ag/S_8_R (*d_s_* = 2.65 nm) nanoparticles versus the bimodal distribution of size of Ag/S_1_G (*d_s_* = 4.6 nm; *d_s_* = 12.5 nm) and Ag/S_1_R (*d_s_* = 6.6 nm; *d_s_* = 11.2 nm). These *K_app_* values are of the same order than those previously reported for AgNPs supported on S-layer/poliuretane [28]. The great influence of the Ag nanoparticles size on *K_app_* is particularly noteworthy when Ag/S_1_V (5.40 h^−1^) and Ag/S_8_V (8.05 h^−1^), having metallic dispersions (*D*%) of 4.37 and 3.22, respectively, were used as catalysts. The mean particle size of the Ag/S_1_V (*d_s_* = 25.24 nm) and Ag/S_8_V (*d_s_* = 36.9 nm) catalysts are notably higher than those of the other AgNP catalysts described above. The large size and the very low dispersion reduce the exposed reactive area of silver to the *p*-NP molecules, thus reducing the active sites performance, which finally leads to a good conversion but with a very low speed. The higher efficiency of the active sites of Ag/S_1_G, Ag/S_8_G, Ag/S_1_R and Ag/S_8_R catalysts are evidenced by the TOF values (11 h^−1^ and 8.9 h^−1^ for Ag/S_1_G and Ag/S_1_R; 11.2 h^−1^and 12 h^−1^ for Ag/S_8_G and Ag/S_8_R) (Table 1).

**Figure 6:**
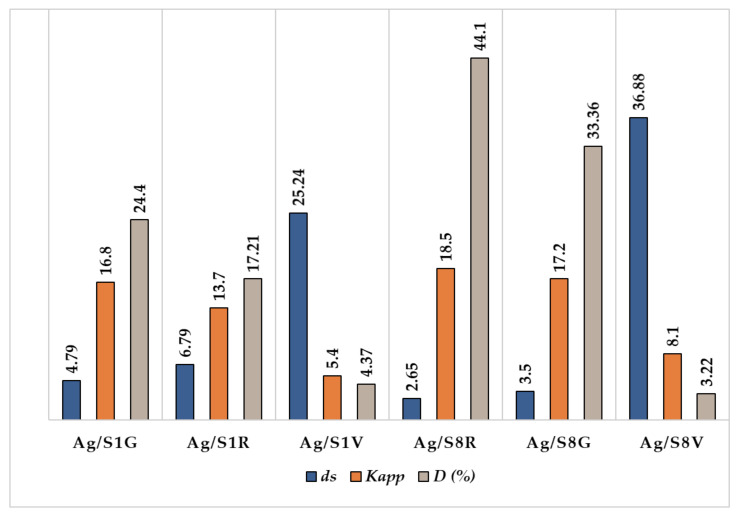
Influence of the reduction procedure on AgNP size (*d_s_*), metal dispersion *D* (%) and *K_app_* for Ag/S_1_G, Ag/S_1_R, Ag/S_1_V, Ag/S_8_G, Ag/S_8_R and Ag/S_8_V catalysts.

All these findings indicate that the strategy applied to reduce Ag ions (pure H_2_, H_2_ mixed with low concentration of NaBH_4_, and a high concentration of NaBH_4_) has a remarkable influence on the performance of the catalysts, since the increment in the AgNP size accompanied with a reduction of the metallic dispersion, leads to an important decrease in *K_app_*, as depicted in Figure 6.

### 3.4. Theoretical Calculations

In order to find a deeper explanation on how the different reduction procedures employed to obtain AgNPs of different sizes that led to catalytic systems having different activities, the substrate-AgNP surface interaction was studied performing molecular calculations at DFT level. One of the most important properties to consider when studying metal catalysts are the geometric and electronic structures of the metal particles, which are governed by their size. For some noble metals, properties such as the work function, for example, are strongly influenced by the size of the particle. When the number of atoms of the metallic nanoparticle increases above 40 (sizes around 1 nm) the gap between HOMO and LUMO becomes smaller compared to that observed in subnanometric particles. For metallic nanoparticles greater than 2 nm, a practically continuous level of energy is formed [58]. Considering that all the AgNP synthetized in this work presented sizes greater than 2 nm, an infinite slab was used for modeling the metallic surface.

Noble metal nanoparticles with FCC (face-centered cubic) crystalline structure preferentially exhibit faces with Miller indices (111) and (100) because they present the lowest energies. The surface of AgNPs was modelled from 4 × 4 cells with three layers of atoms, for both (111) and (100) faces. Additionally, as mentioned above and in accordance with previous studies published by other authors [59,60], the possibility of the existence of both *p*-nitrophenol and *p*-nitrophenolate species was considered, regarding the pH of the medium in which the reaction occurs.

**Figure 7:**
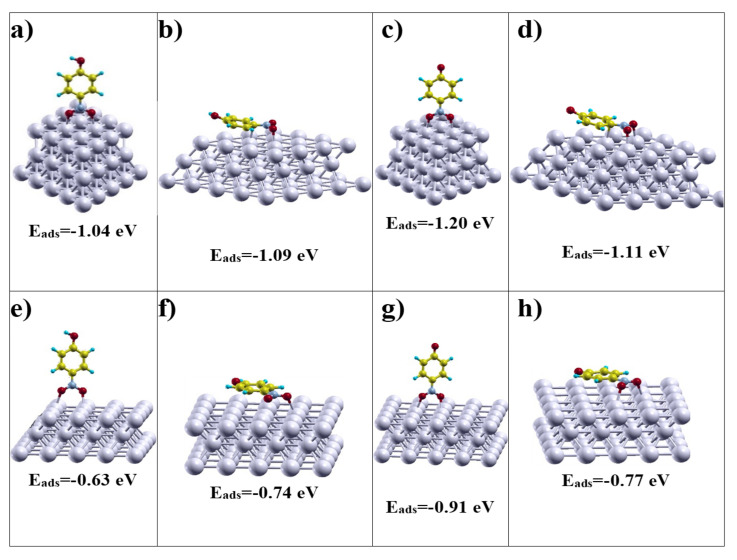
Optimized geometries and adsorption energy (***E_ads_***) for (**a**) *p*-nitrophenol perpendicular on Ag (111), (**b**) *p*-nitrophenol flat on Ag (111), (**c**) *p*-nitrophenolate perpendicular on Ag (111), (**d**) *p*-nitrophenolate perpendicular on Ag (111), (**e**) *p*-nitrophenol perpendicular on Ag (100), (**f**) *p*-nitrophenol perpendicular on Ag (100), (**g**) *p*-nitrophenolate perpendicular on Ag (100) and (**h**) *p*-nitrophenolate perpendicular on Ag (100).

According to the aforementioned information, the adsorption of both *p*-nitrophenol and *p*-nitrophenolate was modelled on (111) and (100) faces, parallel (flat adsorption through the π orbitals of the aromatic system) and perpendicular (through the oxygen atom of the nitro group) to the surface. The geometries of the optimized structures of all the studied systems are shown in Figure 7. As can be seen, the adsorption energies are negative for all the cases, which means that, regardless of the face, the species being adsorbed or the mode of adsorption, all adsorptions are thermodynamically stable. When comparing the adsorption energy on both faces, it can be seen that the adsorption on (111) face is more stable than the adsorption on (100) face, regardless of the substrate species and the adsorption mode. While all the adsorption energies calculated for (111) face exceed the value of –1 eV, those corresponding to (100) face are between –0.63 and –0.91 eV.

On the other hand, the adsorption energies as a function of the different adsorption modes reveal that the most stable adsorption mode for *p*-nitrophenol is flat and parallel to the surface for both (111) and (100) faces. However, when the modelled species is *p*-nitrophenolate, the most stable adsorption mode is perpendicular, adsorbed through the two oxygens of the nitro group. The adsorption energy of *p*-nitrophenolate through the nitro group on (111) face is −1.20 eV and on (100) face is −0.91 eV. These results agree with the fact that due to the pH of the reaction medium, the predominant species adsorbed on the metal surface of the catalyst is *p*-nitrophenolate. Moreover, they are also in accordance with the finding that the only product of the reaction is *p*-aminophenol. For this to happen, the substrate must be adsorbed through the nitro group, the O–N bond is activated and subsequently hydrogenated. If the substrate would be adsorbed through the π-system of the aromatic group, the ring would have been hydrogenated giving 4-nitrocyclohexanol, which is not observed experimentally.

Table 2 presents the values of the bond lengths for the N–O bonds of the nitro group of p-nitrophenol and p-nitrophenolate calculated for perpendicular and flat adsorption modes on (111) and (100) faces, as well as those of both free substrates. The distance between the O atoms of the nitro group and the closest surface Ag atom for all the adsorption possibilities studied are also found in the table. As can be seen, for both adsorption modes and for both species, the distance between the O atoms of the nitro group and the closest surface Ag atom, is somewhat smaller for the (111) face than for the (100) face. Thus, while for the (111) face this distance varies between 2.26 and 2.29 A, for the (100) face this value is between 2.33 and 2.43 A. Furthermore, if this distance is compared for the same crystalline face, but considering the two adsorption modes, it is found that in the case of perpendicular adsorption there is a greater approach between the reagent and the metal surface. Regarding the O–N bond lengths, for the free (non-adsorbed) species they are shorter than the O–N bond lengths calculated for the adsorbed species. While these lengths are 1.23 A and 1.25 A for free *p*-nitrophenol and *p*-nitrophenolate, respectively, the two species adsorbed on a (111) face presented O–N lengths between 1.27 and 1.29 A. The values obtained for the adsorption on a (100) face are between 1.27 A and 1.30 A. The stretching of these bonds in the adsorbed species with respect to the free ones is evidence of the weakening of the corresponding bond, which generates its activation for hydrogenation.

## 4. Conclusions

The main conclusions of the present work can be summarized as follows:

✓ Supported AgNPs were synthesized using supported S-layer proteins (SLPs) as bidimensional regularly arranged biotemplates. The nanoparticles were obtained in a simple way, without needing a stabilizer.
✓ By different reduction strategies AgNPs of variable sizes were obtained on two different SLPs. A drastic reduction with NaBH_4_ led to large AgNPs (between 25 and 37 nm) whereas a smooth reduction with either H_2_ or H_2_/NaBH_4_ at low concentration, led to small AgNPs (sizes between 2 and 7 nm).
✓ Conversion values between 75% and 80% of *p*-NP were observed for all the AgNPs tested, regardless of the average particle size of the NPs. Conversely, the apparent rate constant (*K_app_*) and TOF values were higher for Ag/S_8_R and Ag/S_8_G, the systems showing the smallest particle size.
✓ Theoretical results confirmed the stretching of the N-O bond, meaning that it is activated for the hydrogenation reaction.
✓ The most favored, thermodynamically stable adsorption mode of *p*-nitrophenolate species is through the nitro group, which would ensure *p*-aminophenol as the only feasible product of the reaction, which was corroborated experimentally.
✓ Finally, obtaining AgNPs supported on a biological system such as LSP with outstanding catalytic activity is an interesting and environmentally friendly contribution, both with respect to its obtaining mechanism and also regarding the elimination of a dangerous pollutant such as *p*-NP.

## Figures and Tables

**Table 1:** Metal particle size (*d_s_*), dispersion (*D*), apparent rate constant (*K_app_*) and turnover frequency (TOF) for the studied catalysts.

Catalyst	Kapp (h^−1^)	*d_s_* (nm) *	*D* (%)	TOF (mol *p*-NP/mol Ag × h)
Ag/S_1_G	16.8	4.79	24.41	11.0
Ag/S_1_R	13.7	6.79	17.21	8.9
Ag/S_1_V	5.4	25.24	4.37	3.3
Ag/S_8_G	17.7	3.5	33.36	11.2
Ag/S_8_R	18.47	2.65	44.10	12.0
Ag/S_8_V	8.1	36.88	3.22	5.2

* Average value.

**Table 2:** Calculated O–Ag (***l*_O–Ag_**) and O–N (***l*_O–N_**) bond length and adsorption energies (**E_ads_**).

Crystalline Face	Substrate Species	Adsorption Mode	*l*_O–Ag_ (A)	*l*_O–N_ (A)	E_ads_ (eV)
(111)	*p*-nitrophenol	perpendicular	2.26	1.27	−1.04
		flat	2.29	1.29	−1.09
	*p*-nitrophenolate	perpendicular	2.27	1.28	−1.20
		flat	2.29	1.29	−1.11
(100)	*p*-nitrophenol	perpendicular	2.33	1.28	−0.63
		flat	2.44	1.27	−0.74
	*p*-nitrophenolate	perpendicular	2.34	1.30	−0.91
		flat	2.43	1.28	−0.69
-	*p*-nitrophenol	-	-	1.23	-
-	*p*-nitrophenolate	-	-	1.25	-
